# Predictive value of bronchoscopy combined with CT score for refractory mycoplasma pneumoniae pneumonia in children

**DOI:** 10.1186/s12890-024-02996-w

**Published:** 2024-05-22

**Authors:** Weihong Lu, Xiangtao Wu, Yali Xu, Tuanjie Wang, Aiju Xiao, Xixia Guo, Yuping Xu, Duoduo Li, Shujun Li

**Affiliations:** Department of Pediatrics, the First Affiliated Hospital of Xinxiang Medical University, No. 88 of Jiankang Road, Weihui, Henan province 453100 China

**Keywords:** Mycoplasma pneumoniae pneumonia, Computed tomography score, Bronchitis score, Bronchoalveolar lavage, Children

## Abstract

**Introduction:**

Mycoplasma pneumoniae pneumonia (MPP) is prevalent in paediatric patients and can progress to refractory mycoplasma pneumoniae pneumonia (RMPP).

**Objective:**

To assess the predictive value of bronchoscopy combined with computed tomography (CT) score in identifying RMPP in children.

**Methods:**

A retrospective analysis was conducted on 244 paediatric patients with MP, categorising them into RMPP and general mycoplasma pneumoniae pneumonia (GMPP) groups. A paired t-test compared the bronchitis score (BS) and CT score before and after treatment, supplemented by receiver operating characteristic (ROC) analysis.

**Results:**

The RMPP group showed higher incidences of extrapulmonary complications and pleural effusion (58.10% and 40%, respectively) compared with the GMPP group (44.60%, *p* = 0.037 and 18.71%, *p* < 0.001, respectively). The CT scores for each lung lobe were statistically significant between the groups, except for the right upper lobe (*p* < 0.05). Correlation analysis between the total CT score and total BS yielded *r* = 0.346 and *p* < 0.001. The ROC for BS combined with CT score, including area under the curve, sensitivity, specificity, and cut-off values, were 0.82, 0.89, 0.64, and 0.53, respectively.

**Conclusion:**

The combined BS and CT score method is highly valuable in identifying RMPP in children.

## Introduction

*Mycoplasma pneumoniae* pneumonia (MPP) is notably prevalent in paediatric wards, accounting for approximately 10–40% of paediatric community-acquired pneumonia pathogens [1]. This condition can cause not only respiratory tract infections but also lead to extrapulmonary manifestations, such as myocardial damage, Stevens–Johnson syndrome, encephalitis, and liver damage [2, 3]. More concerning is that *mycoplasma pneumoniae (MP)* can result in refractory mycoplasma pneumoniae pneumonia (RMPP).

Patients treated for MPP who exhibit persistent fever, worsening clinical signs, and deteriorating pulmonary imaging findings, as well as extrapulmonary complications, are diagnosed with RMPP. The early administration of appropriate anti-mycoplasma drugs is vital. Although macrolides are the standard treatment for mycoplasma infections, the incidence of macrolide-resistant *mycoplasma pneumoniae* infections in children is increasing [4]. Glucocorticoids have been shown to alleviate excessive immune response and aid lung recovery in RMPP [5]. However, numerous RMPP cases do not respond to glucocorticoid treatment. Some researchers believe that the formation of sputum emboli is a key pathological manifestation of this poor therapeutic response [6]. Fibreoptic bronchoscopy (FOB) and bronchoalveolar lavage (BAL) procedures have shown substantial efficacy in treating pneumonia, particularly in cases involving sputum thrombi [7, 8]. Early intervention and assessment are crucial for improving MPP outcomes, making it essential to identify more efficient assessment indicators for RMPP. Current predictors mainly rely on symptoms and laboratory tests [6, 9]. Mycoplasma pneumonia often presents with mild symptoms and severe pulmonary imaging changes, which can lead to delayed or missed diagnoses if based solely on clinical information and serum characteristics. Therefore, establishing more intuitive lung data, such as through FOB and computed tomography (CT) examinations, is necessary for an effective early assessment of pulmonary severity. However, these methods have their limitations: a bronchoscope can only observe lesions in the bronchus or trachea, while CT scans identify inflammation outside the bronchus but do not reveal the tracheal interior. Employing both methods simultaneously enables a more comprehensive lung assessment, compensating for their respective limitations.

Fibreoptic bronchoscopy has been utilised by researchers to assess airway inflammation severity by evaluating changes in secretion volume and colour, resulting in a bronchitis score (BS) [10]. The BS directly quantifies airway lesions in patients with MP infections, offering substantial clinical value. Meanwhile, the CT score is primarily used to assess pulmonary progression in COVID-19 [11]. Mycoplasma pneumoniae pneumonia shares similar pulmonary pathological changes with COVID-19, characterised by interstitial lung lesions, ground-glass opacities, paving stone signs, consolidation, and other typical CT findings in children. However, there is currently no reliable score for assessing the severity of pulmonary inflammation in MPP. Although BS and CT score can effectively assess the disease through direct bronchial and lung signs, each method has its limitations when used individually in evaluating MP. For example, CT imaging may show extensive lung consolidation despite minimal airway secretion, or it may indicate mild lung inflammation but exhibit notable airway lesions. Therefore, combining both approaches is advantageous in evaluating and predicting RMPP. This study retrospectively analysed clinical data from 244 children with MPP to examine the value of combining BS and CT score in quantifying MPP pulmonary conditions and predicting RMPP.

## Research methods

### Case definition and identification

In this study, 244 patients with MP admitted to the Department of Pediatrics at the First Affiliated Hospital of Xinxiang Medical University between January 2019 and December 2021 were retrospectively analysed.

The inclusion criteria included the following: (1) patients meeting the diagnostic criteria for pneumonia; (2) *mycoplasma pneumoniae* infection confirmed as positive via polymerase chain reaction in the nasopharyngeal swab, sputum, or alveolar lavage fluid, or a mycoplasma pneumoniae antibody titre in the blood of ≥1:160^6^; (3) indications for BAL such as radiographically proven large lung lesions, lung consolidation, and atelectasis, determined at the attending physician’s discretion.

The exclusion criteria included the following: (1) diseases caused by other pathogens; (2) aspiration of foreign bodies; (3) hospital-acquired pneumonia; (4) chronic respiratory diseases (e.g. bronchiectasis, asthma, confirmed or suspected active tuberculosis); (5) malignant tumours, solid organ transplantation or surgery, immune deficiency, or use of immunosuppressive drugs; (6) congenital, inherited metabolic diseases and other underlying diseases; (7) intolerance to BAL; (8) refusal of participation by the patient’s parents or guardians; (9) patients with incomplete information.

Regarding the diagnostic criteria for RMPP [9], patients with MP who had been treated with macrolide antibiotics for 7 days or more and continued to exhibit fever, clinical signs, worsening pulmonary imaging findings, and extrapulmonary complications were classified as having RMPP. This study received approval from the Ethics Committee of the First Affiliated Hospital of Xinxiang Medical University (ethics number: 2,020,252).

### Study design

Following the diagnostic criteria for refractory mycoplasma pneumoniae pneumonia, the patients were placed into either the RMPP group or the general *mycoplasma pneumoniae* pneumonia (GMPP) group. For each participant, the following data was collected and analysed: demographic details, fever, coughing, wheezing, onset of MP clinical manifestations, laboratory test results, chest imaging before and after treatment, CT grade, and endoscopic BS. Furthermore, receiver operating characteristic (ROC) analysis of the CT score and BS was conducted to assess their predictive value for RMPP.

Both groups received macrolide antibiotics intravenously for 1–2 weeks; glucocorticoids were administered to most patients at a dosage of 1–2 mg/kg/day intravenously for 3–5 days. Some patients with RMPP or severe diseases were given medication for a longer duration as deemed appropriate. The indicators for glucocorticoid application included shortness of breath, dyspnoea, and increased respiratory secretions; persistent symptoms of toxicity such as high fever and poor mental state despite regular antibiotic treatment; and chest CT indicating solid lung lesions or no improvement in the original lesions.

### Fibreoptic bronchoscopy and bronchoalveolar lavage procedures

Fibreoptic bronchoscopy and BAL procedures were performed when CT scans revealed solid lung lesions, prolonged disease duration combined with unclear pathogens, and poor response to medical treatment. BAL samples were collected from the lung segment, showing the most severe lesions on lung CT examination. The lavage solution, comprising sterile saline heated to 37 °C, was infused three to five times, with each infusion being 1 ml/kg, followed by immediate aspiration. The saline was aspirated under a negative pressure of 6.65–13.3 kPa (50–100 mmHg). A minimum of 40% of the BAL fluid was recovered. Following this, the infusion of macrolide antibiotics and steroid therapy continued. A second BAL was considered if there was worsening pulmonary consolidation after 1 week. If sputum clots or plastic bronchitis were identified during the first BAL, 1–2 additional BAL procedures were conducted as appropriate, depending on the treatment’s effectiveness. A second BAL was also considered in cases meeting the RMPP diagnosis criteria. However, most patients exhibiting a single BAL showed substantial improvement in symptoms or pulmonary consolidation following the initial procedure.

#### Endoscopic bronchitis score

The FOB process was conducted using an Olympus 3.1 mm BF-XP290 or Olympus 4.2 mm BF-P290 bronchoscope under the supervision of the treating physician. Endoscopic video recordings were independently reviewed and scored by two experienced endoscopists who were blinded to the patients’ clinical history. Scoring sites included the trachea, right main trunk, right upper lobe, right middle trunk of the bronchus, right middle lobe, right lower lobe, left main bronchus, left upper lobe (including lingual lobe), and left lower lobe. Each site was assessed for six bronchoscopic visual features: the amount and colour of secretions and the presence or absence of oedema, swelling, erythema, and pallor of the airway mucosa. The colour of secretions was scored according to the BronkoTest® sputum colour chart, ranging from 0 to 8. The amount of secretions in the bronchial lumen is measured on a scale of 1 to 6, with a higher score indicating the presence of more secretions. In the first scoring round, features such as mucosal oedema, swelling, erythema, and pallor were graded on a scale of 0 to 2 based on severity (0 = none, 1 = slight, 2 = moderate to severe). In the second correction round, a composite score of 0 to 3 was assigned for each mucosal appearance site based on the number of affected sites (0 = none; 1 = one point for less than half of the nine sites; 2 = more than half of the nine sites scored one point, or less than half scored two points; 3 = more than half of the nine sites scored more than two points) [9]. In cases of considerable deviation, a senior physician re-evaluated the scores.

#### Computed tomography score

Computed tomography scores were determined based on the extent of ground-glass opacification across the lobes, with a maximum possible score of 5 for each of the five lobes. The scoring criteria were as follows [11]: 0 = no involvement; 1 = less than 5% involvement; 2 = 5–25% involvement; 3 = 26–49% involvement; 4 = 50–75% involvement; and 5 = more than 75% involvement. Different weights were assigned to the three types of CT findings: ground-glass opacity (GGO), paving stone sign, and consolidation. The basal CT score was increased by 1 point if a paving-stone sign was present in one lobe and by 2 points if consolidation (with or without a paving-stone sign) was observed. The total CT score, ranging from 0 to 35, represented the cumulative scores for each of the five lobes, with the highest score indicating consolidation in all five lobes.

### Statistical analysis

The statistical analysis was carried out using IBM SPSS Statistics software, version 23. The Kolmogorov–Smirnov test determined whether continuous variables adhered to a normal distribution. Where this was the case, the data were presented as mean ± standard deviation. Alternatively, for non-normally distributed data, the presentation was in terms of the median. The unpaired t-test facilitated the comparison of two independent variables, while a one-way analysis of variance (ANOVA) was employed for comparing multiple continuous variables. Pearson’s correlation test assessed the relationship between the CT score and BS. A paired t-test was utilised to compare pre- and post-treatment scores. Categorical variables were described using frequency (percentage) and compared using the chi-squared test. The ROC curve evaluated the predictive capacity of the endoscopic BS and CT score in relation to RMPP. Both univariate and multivariate logistic regression analyses identified the independent contributions of BS and CT score in predicting RMPP. Statistical significance was established at *p* ≤ 0.05.

## Results

### Study procedure

**Fig. 1:**
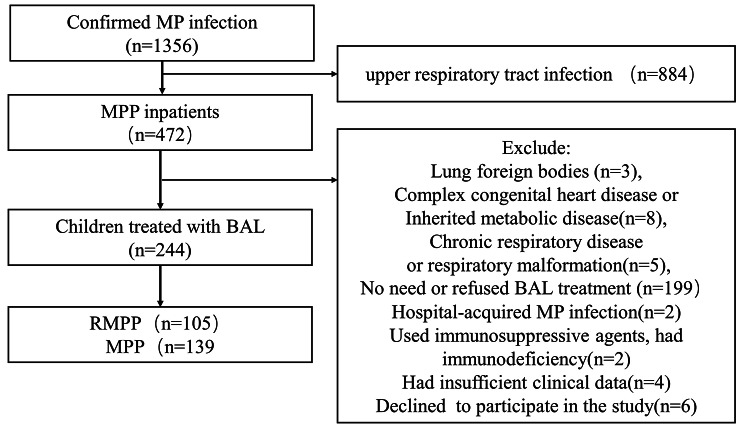
Flow chart of the study. (MP: mycoplasma pneumonia; RMPP: refractory mycoplasma pneumoniae pneumonia; GMPP: general mycoplasma pneumoniae pneumonia; BAL: bronchoalveolar lavage)

Between January 2019 and December 2021, a total of 1,356 patients were diagnosed with *mycoplasma pneumoniae* infection at our hospital, including 472 who were hospitalised. Excluded from the study were patients with lung foreign bodies (*n* = 3), combined complex congenital heart disease or inherited metabolic disease (*n* = 8), chronic respiratory disease or respiratory malformation (*n* = 5), those who did not require or refused BAL treatment (*n* = 199), cases of hospital-acquired *mycoplasma pneumoniae* infection (*n* = 2), patients using immunosuppressive agents or with immunodeficiency (*n* = 1), those with insufficient clinical data (*n* = 4), and those who declined to participate in the study (*n* = 6). Consequently, 244 patients were enrolled. Of these, 105 had RMPP, and 139 had GMPP, as illustrated in Fig. 1.

### Clinical, computed tomography and fibreoptic bronchoscopy characteristics across the two groups

There was no significant difference in gender distribution between the two groups (*p* = 0.301). The average age in the RMPP group was 5.84 ± 2.86 years, which was significantly older than in the GMPP group (*p* = 0.002). Symptoms of dyspnoea, including wheezing and shortness of breath, were observed in 31.7% of patients in the GMPP group, compared with 2.9% in the RMPP group (*p* < 0.001). The incidence of extrapulmonary complications and pleural effusion was higher in the RMPP group (58.1% and 40.0%, respectively) than in the GMPP group (44.6% and 18.7%, respectively) (*p* = 0.037 and < 0.001, respectively). The Acute Physiology and Chronic Health Evaluation (APACHE) II score of the GMPP group (5.38 ± 5.51) exceeded that of the RMPP group (3.05 ± 4.06) (*p* = 0.002), and the intensive care unit occupancy rate was higher in the GMPP group (38.13%) compared with the RMPP group (14.29%) (*p* = 0.027). However, no significant differences were observed in the length of hospital stay or the sequential organ failure assessment score (*p* = 0.353, 0.568).

**Fig. 2:**
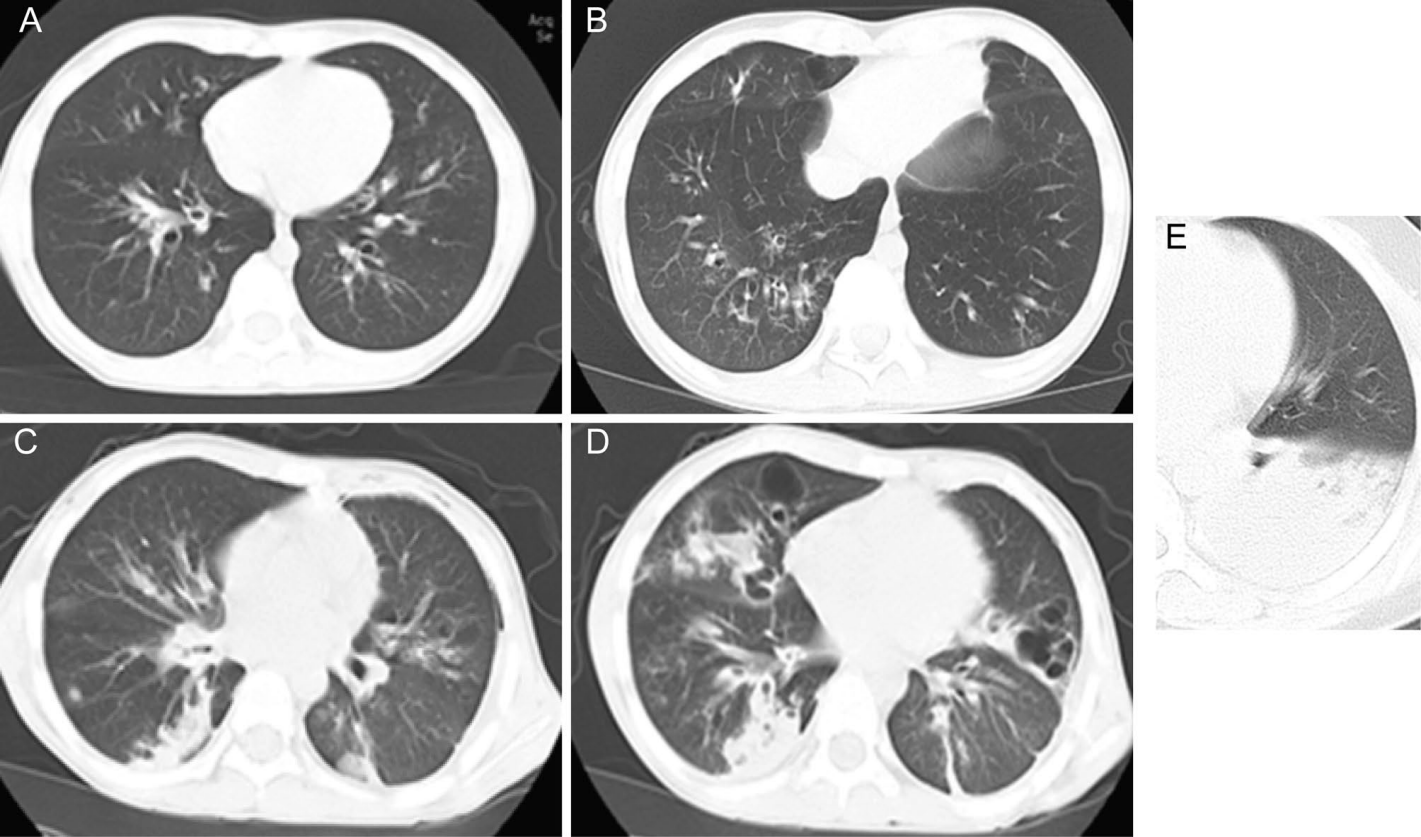
Typical CT images of the CT score. **A**: CT score was 1; **B**: GGO involvement was less than 25%, and CT score was 2 points; **C**: Right inferior lobe of lung: <50%, consolidation, CT score 4 points; Left inferior lobe: GGO, 26–49%, 3 points.; **D**: Right inferior lobe of lung, 50–75% area involved, consolidation, CT score 6 points; E: Left inferior lobe consolidation > 75%, CT score 7 points

Regarding CT scores, the total score and scores for each lung lobe, except the right upper lobe, differed significantly between the two groups (*p* < 0.05). Representative CT images are presented in Fig. 2. In terms of endoscopic findings, the probability of shaping and sputum thrombolysis was notably higher in the RMPP group compared with the GMPP group (*p* < 0.001). The secretion volume and colour value were also greater in the RMPP group (*p* = 0.007, 0.028), while no significant differences were found in other scores (*p* > 0.05). The MP positive rate was significantly higher in the RMPP group (*p* < 0.001), as detailed in Table 1.

**Table 1:** Clinical, CT, and FOB characteristics between the RMPP and GMPP groups

Variables	RMPP (*n* = 105)	GMPP (*n* = 139)	*P* value
General information			
Age, years	5.84 ± 2.86	4.46 ± 3.89	0.002
Male, n	61 (58.1)	71 (51.1)	0.301
Clinical characteristics			
History before admission, days	10.95 ± 5.33	13.11 ± 19.8	0.220
Fever, days	10.49 ± 5.45	8.70 ± 13.25	0.153
Cough, days	8.95 ± 6.08	10.68 ± 18.49	0.304
Dyspnea, n	3 (2.9)	44 (31.7)	< 0.001
APACHE II	3.05 ± 4.06	5.38 ± 5.51	0.002
SOFA	4.05 ± 3.40	4.46 ± 2.70	0.568
Length of hospital stay, days	14.70 ± 7.13	15.75 ± 10.36	0.353
ICU, n	15 (14.29)	53 (38.13)	0.027
CT score			
Total score	13.65 ± 4.10	9.65 ± 3.61	< 0.001
Right upper lobe	2.64 ± 2.57	2.19 ± 2.03	0.140
Right middle lobe	2.93 ± 2.46	1.96 ± 1.82	0.001
Right lower lobe	2.90 ± 2.49	2.14 ± 2.12	0.013
Left Upper lobe	2.06 ± 2.17	1.33 ± 1.65	0.005
Left lower lobe	3.11 ± 2.72	2.05 ± 2.17	0.001
Laboratory tests			
BAL MP positive, n	91 (86.7)	94 (67.6)	0.001
A throat swab was positive for MP, n	58 (55.2)	39 (28.1)	< 0.001
MP DNA, copies/ml	1.82E + 08 ± 5.14E + 08	2.97E + 07 ± 1.40E + 08	0.001
Immunoglobulin abnormalities, n	18 (17.1)	22 (15.8)	0.783
Abnormal lymphocyte subsets (NK), n	13 (12.4)	29 (20.9)	0.082
Endoscopic BS			
Amount of secretion	4.98 ± 0.98	4.65 ± 0.92	0.007
Color of secretion	2.65 ± 0.83	2.40 ± 0.95	0.028
Mucosal edema	1.18 ± 0.76	1.27 ± 0.79	0.395
Mucosal eminence	0.14 ± 0.47	0.19 ± 0.64	0.553
Mucosal erythema	0.87 ± 0.61	0.86 ± 0.73	0.970
Mucosal pale	0.43 ± 0.65	0.47 ± 0.66	0.646
Total endoscopic score	11.89 ± 2.27	9.81 ± 2.80	< 0.001
Plastic bronchitis, n	36 (34.3)	18 (12.9)	< 0.001
Times of BAL	1.47 ± 0.80	1.55 ± 1.58	0.605
Complications			
Extrapulmonary complications, n	61 (58.1)	62 (44.6)	0.037
Pleural effusion, n	42 (40.0)	26 (18.7)	< 0.001
Cardiovascular system abnormalities, n	10 (9.5)	19 (13.7)	0.322
Abnormal liver function, n	15 (14.3)	11 (7.9)	0.110
Anemia, n	4 (3.8)	9 (6.5)	0.359
Urinary system abnormalities, n	10 (9.5)	16 (11.5)	0.618
Systemic inflammatory response syndrome, n	13 (12.4)	16 (11.5)	0.835
Abnormal coagulation function, n	4 (3.8)	2 (1.4)	0.407
Death, n	1 (1.0)	1 (0.7)	1.000

Variables were expressed as *n* (%) or mean ± standard deviation. APACHE II: Acute Physiology and Chronic Health Evaluation II; SOFA: sequential organ failure assessment; BAL: bronchoalveolar lavage

### Correlation analysis of total computed tomography score, total bronchitis score, and *mycoplasma pneumoniae*-DNA sequence copies

**Fig. 3:**
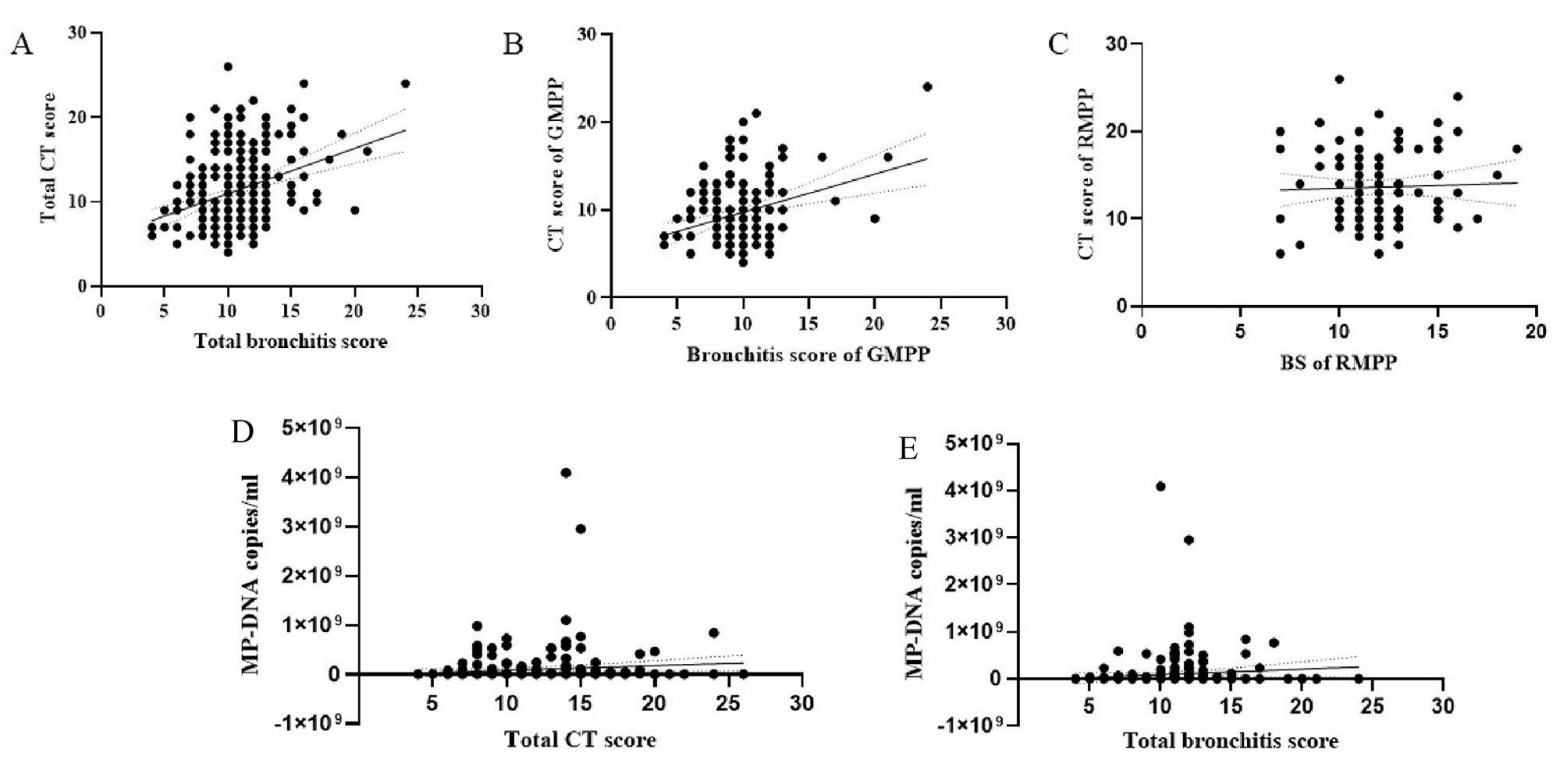
Correlation analysis between CT score and BS **A**: Total BS score vs. total CT score, *r* = 0.346, *P* < 0.001; **B**: GMPP group, BS total score vs. CT total score, *r* = 0.338,*P* < 0.001; **C**: BS total score vs. CT total score, *r* = 0.037, *P* = 0.7078.D: BS total score vs. MP-DNA sequence copies, *r* = 0.09, *P* = 0.16; E: CT total score vs. MP-DNA sequence copies, *r* = 0.13, *P* = 0.079. (BS: bronchitis score)

The pairwise correlation analysis of the total CT score, total BS, and *mycoplasma pneumoniae*-DNA sequence copies yielded the following results: total BS score versus total CT score, *r* = 0.346, *p* < 0.001; total BS score versus *mycoplasma pneumoniae*-DNA sequence copies, *r* = 0.09, *p* = 0.16; and total CT score versus *mycoplasma pneumoniae*-DNA sequence copies, *r* = 0.13, *p* = 0.079. In the RMPP group, *r* = 0.0370 and *p* = 0.7078, while in the GMPP group (total BS score and total CT score), *r* = 0.338 and *p* < 0.001 (Fig. 3).

### Predictive effect of bronchitis score on refractory mycoplasma pneumonia

**Fig. 4:**
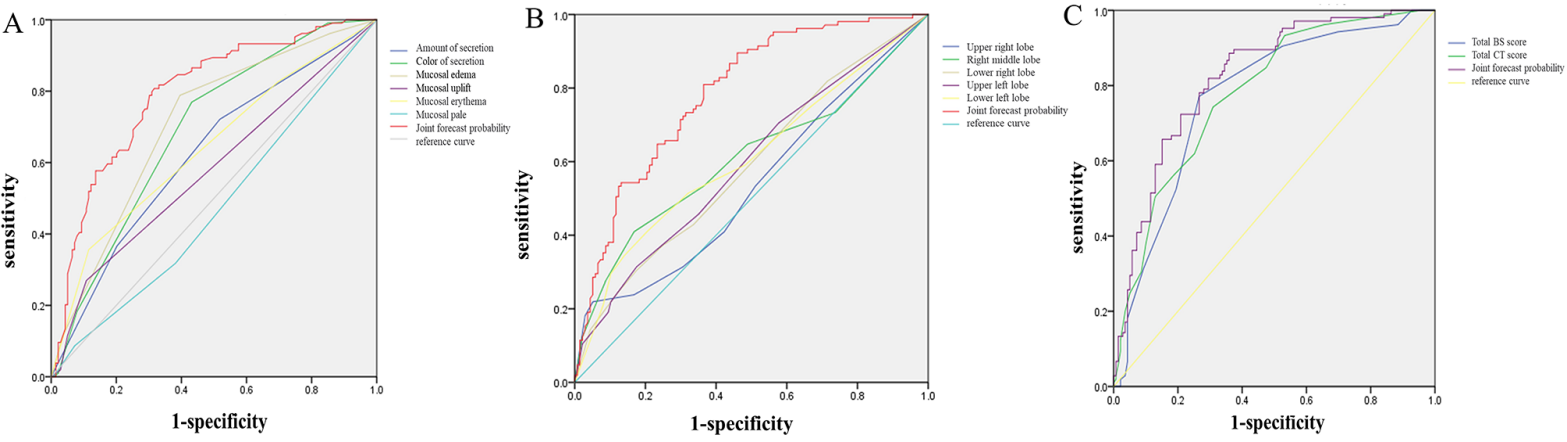
**A** ROC curve of the predictive effect of BS on RMPP (ROC: receiver operating characteristic). **B** ROC curve of CT score of each lung lobe for predicting RMPP: AUC = 0.786, sensitivity 0.81, specificity 0.64, cut-off value 0.44, *P* = 0.000. (AUC: area under the curve). **C** ROC curve of BS, CT total score and BS/CT combined prediction in RMPP patients had better prediction effect than the single prediction, with AUC of 0.824, sensitivity of 0.89, specificity of 0.64, cut-off value of 0.53, *P* = 0.000

The BS components, including secretion volume, colour, oedema, swelling, and erythema, had high predictive values (AUC = 0.622, 0.691, 0.709, 0.579, 0.641, respectively; *p* < 0.05), with the exception of mucosal pallor (AUC = 0.473, *p* = 0.468). The highest sensitivity was observed for secretion volume (0.72), colour (0.77), and mucosal oedema (0.79), while the highest specificity was noted for mucosal uplift (0.89) and erythema (0.88); moreover, pale mucosa demonstrated high specificity (0.93). The AUC, sensitivity, specificity, and cut-off values for the combined prediction of these six indicators were 0.793, 0.81, 0.68, and 0.49, respectively (*p* < 0.001), indicating that the BS’s assessment of secretions and mucosa has a considerable predictive effect on RMPP (Fig. 4A), as detailed in Table 2.

**Table 2:** ROC analysis of bronchitis score on RMPP

	AUC	Std. Error	*P* value	95% confidence interval	sensitivity	specificity	cutoff value
Amount of secretion	0.622	0.036	0.001	0.551–0.693	0.72	0.48	0.20
Color of secretion	0.691	0.034	0.000	0.625–0.757	0.77	0.57	0.34
Mucosal edema	0.709	0.033	0.000	0.643–0.774	0.79	0.60	0.39
Mucosal eminence	0.579	0.038	0.035	0.505–0.653	0.27	0.89	0.16
Mucosal erythema	0.641	0.036	0.000	0.570–0.712	0.36	0.88	0.24
Mucosal pale	0.473	0.037	0.468	0.399–0.546	0.09	0.93	0.01
Collaborative forecasting	0.793	0.029	0.000	0.736–0.850	0.81	0.68	0.49

### Predictive value of computed tomography score in refractory mycoplasma pneumoniae pneumonia

The specificity of the CT score for the five lobes was ≥ 0.8, but the sensitivity was low at < 0.5 (Table 3). Except for the right upper lobe, the ROC analysis of the other four lobes showed statistical significance (*p* < 0.05); however, the AUC for a single lobe’s prediction was approximately 0.6. The combined predictive value for all lobes was substantially improved, with an AUC of 0.786, sensitivity of 0.81, specificity of 0.64, and a cut-off value of 0.44 (*p* < 0.001) (Table 3; Fig. 4B). When combined with the CT scores (Table 2), it was apparent that the right upper lobe was the lobe with multiple *mycoplasma pneumoniae* infections, showing little difference between the two groups. The left upper lobe had the lowest score, indicating a mild degree of *mycoplasma pneumoniae* infection, while the left lower lobe had the highest mean score and was most indicative of RMPP.

**Table 3:** ROC analysis of CT scores of each lung lobe for RMPP

	AUC	Std. Error	*P* value	95% confidence interval		Sensitivity	Specificity	Cut-off value
Right upper lobe	0.536	0.038	0.340	0.461	0.610	0.22	0.95	0.17
Right middle lobe	0.607	0.038	0.004	0.533	0.682	0.41	0.83	0.24
Right lower lobe	0.588	0.037	0.019	0.516	0.661	0.30	0.82	0.13
Left Upper lobe	0.593	0.037	0.013	0.520	0.665	0.31	0.82	0.14
Left lower lobe	0.608	0.037	0.004	0.535	0.681	0.41	0.80	0.21
Collaborative forecasting	0.786	0.029	0.000	0.729	0.843	0.81	0.64	0.44

### Comparison of the receiver operating characteristic of bronchitis score, CT score, and the combination of both

The effects of the BS and CT score on predicting RMPP were as follows: the total AUC for the BS was 0.771, and that for the CT score was 0.781, with both *p*-values being less than 0.001. The sensitivity and specificity for the BS (0.77, 0.73) were higher than those for the CT score (0.74, 0.69), and the cut-off value was 0.51. For the combined BS/CT prediction, the AUC, sensitivity, specificity, and cut-off values were 0.824, 0.89, 0.64, and 0.53, respectively (*p* < 0.001) (refer to Table 4; Fig. 4C).

**Table 4:** ROC curve analysis of BS, CT total score and BS/CT combined prediction

	AUC	Std. Error	*P* value	95% confidence interval	Sensitivity	Specificity	Cut-off value
BS	0.771	0.031	0.000	0.711 to 0.831	0.77	0.73	0.51
CT score	0.781	0.029	0.000	0.723to 0.838	0.74	0.69	0.43
Collaborative forecasting	0.824	0.026	0.000	0.722 to 0.876	0.89	0.64	0.53

### Univariate and multivariate analyses

Univariate and multivariate logistic regression analyses were performed to ascertain the independent roles of BS and CT score in predicting RMPP (see Table 5). In the univariate analysis, the BS, CT score, and age were all significantly associated with the presence of RMPP (*p* < 0.003 for each). In the multivariate analysis, both the BS (odds ratio [OR]: 1.293, 95% confidence interval [CI]: 1.125–1.487, *p* < 0.001) and the CT score (OR: 1.291, 95% CI: 1.184–1.409, *p* < 0.001) emerged as independent predictors of RMPP, irrespective of age (OR: 1.177, 95% CI: 1.074–1.291, *p* = 0.001).

**Table 5:** Univariable and multivariable analyses for the presence of RMPP

	Univariable analysis		Multivariable analysis	
Variables	OR (95% CI)	*P* value	OR (95% CI)	*P* value
BS	1.402 (1.236–1.591)	< 0.001	1.293 (1.125–1.487)	< 0.001
CT score	1.302 (1.202–1.410)	< 0.001	1.291 (1.184–1.409)	< 0.001
Age, years	1.119 (1.039–1.206)	0.003	1.177 (1.074–1.291)	0.001

BS: bronchitis score; CI: confidence interval; OR: odds ratio; RMPP: refractory mycoplasma pneumoniae pneumonia

## Discussion

The BS demonstrated a high predictive value for aspects such as secretion volume, colour, oedema, swelling, and erythema. The CT score exhibited specificity greater than 0.8 for all five lobes, but its sensitivity was below 0.5. Combining these two scores enhances the overall predictive value by compensating for their respective limitations in specificity and sensitivity.

The study found that patients in the GMPP group exhibited more severe clinical manifestations, including wheezing and shortness of breath. However, this group experienced less severe extrapulmonary complications and pleural effusion compared with the RMPP group. This distinction might account for the minor differences observed in the duration of hospital stays between the two groups. These findings align with those reported by Zhang [12] and Poddighe [13] and are further supported by Lee et al., who observed fewer severe cases in their RMPP group compared with their GMPP group [2]. Such consistency with previous studies underscores the relevance of these results for early identification of RMPP [14]. Nonetheless, there is still a need for effective auxiliary examination indicators to predict the severity of the condition.

Previous research on predicting outcomes of MPP and assessing disease severity has primarily focused on serological indicators, such as lactate dehydrogenase, interleukin-6, procalcitonin, white blood cell count, and D-dimer [6]. Cheng et al. identified lactate dehydrogenase, albumin, neutrophil ratio, and high fever as important predictors of RMPP [15]. Conversely, Choi et al. highlighted peak body temperature, neutrophil ratio, platelet count, interleukin-6, lactate dehydrogenase, and atelectasis as key predictors [16]. Currently, few radiological scoring tools designed for the respiratory system are applicable to children with MP [17]. Among the various radiological scoring tools for COVID-19 [11, 18], a standard tool that considers both the degree of pulmonary lobe involvement and changes in CT results has been developed to assess COVID-19 pneumonia more comprehensively through continuous chest CT examinations. This scoring method is also suitable for MP cases with interstitial changes. This study is notable as the first to apply this CT scoring method to the chest imaging assessment of MP and is among the few to quantify CT changes in predicting MPP outcomes. Yan and Huang et al. focused solely on the influencing factors of imaging delay and did not evaluate the severity of the imaging [9, 19].

The one-way ANOVA results indicated differences in CT scores among the five lobes in the RMPP group, but there was no notable difference among the lobes in the GMPP group. In understanding the pathogenesis of MPP, evaluating changes in the degree of pulmonary lobe involvement and CT findings emerges as crucial for assessing the clinical progression and regression of MP. This study utilised quantitative scores to more accurately conclude that in children with RMPP, the left upper lung shows the least involvement, whereas the left lower lung shows the most. These insights help to summarise the conditions of different MPP lobes [8]. Additionally, ROC analysis of the CT scores suggested that these scores could also predict RMPP. This method holds promising application prospects for MPP infection, indicating its potential as a useful tool for doctors in assessing the risk of RMPP at an early stage.

The severity of pulmonary inflammation should be evaluated using CT, and its therapeutic effects should also be reflected in bronchial and endobronchial inflammation and secretion, the latter being more intuitive. Researchers proposed the bronchitis scoring tool as early as 1989 [20], which was later improved by Thomas et al. in 2018 [21]. A 2020 prospective study found substantial correlations between secretion volume, colour, mucosal oedema, and erythema, with BAL neutrophils indicating the level of airway inflammation [10]. While the BS is primarily used in assessing asthma and chronic obstructive pulmonary disease [22, 23], its application in the quantitative assessment of pulmonary conditions in infectious pneumonia is less common [8, 24]. Hence, this endoscopic scoring standard requires further validation through extensive studies, and there is a paucity of reports on the severity of MP assessed through bronchoscopy for endobronchial changes.

This study is the first to quantitatively evaluate the endoscopy score and CT score in relation to MPP-infected airways. It was found that the incidence of shaping, sputum thrombolysis, secretion volume, and colour depth were higher in the RMPP group than in the GMPP group. Along with pale mucosa, indicators such as discharge quantity, colour, oedema, uplift, and erythema demonstrated higher predictive value. In particular, discharge quantity, colour, and mucosal oedema showed better sensitivity. When combined, these six indicators achieved an area under the curve (AUC) of 0.793, with a sensitivity of 0.81 and a specificity of 0.68. The cut-off value was 0.49 (*p* < 0.001), which could be used as a predictor of RMPP. In this study, FOB and BAL procedures were conducted prior to the RMPP diagnosis. The current diagnostic criteria apply to children with MPP who exhibit persistent fever, worsening clinical signs, pulmonary imaging findings, and extrapulmonary complications despite over a week’s treatment with macrolide antimicrobials. Both RMPP and GMPP can be treated with FOB, and during this 7-day period, the child may require FOB for treatment. Consequently, using FOB and BAL for prediction aids clinicians in making early judgements.

The CT score, BS, and *mycoplasma pneumoniae*-DNA sequencing utilised in this study are intrinsically linked to MP and provide a more direct reflection of the severity and prognosis of *mycoplasma pneumoniae* infection compared with serological indices. The *mycoplasma pneumoniae* sequence numbers showed no statistical significance in the first two groups, while the correlation analysis in the RMPP group also yielded no substantial results. This outcome may be linked to the rising incidence of *mycoplasma pneumoniae* resistance mutations [25], although it should be noted that the number of cases in this study was relatively small.

This research is pioneering in its combination of BS and CT score to predict RMPP. While Zhao et al. identified cases of plastic bronchitis through tracheoscopy as a means to predict RMPP, their study did not involve quantitative scoring [6]. Moreover, Huang et al. explored the relationship between clinical, laboratory, radiological, and bronchoscopic studies and the prognosis of fungal pneumonia [26], and Yan et al. established a link between corticosteroid resistance, timing of interventional bronchoscopy, atelectasis, and mucous thrombi formation with delayed remission in RMPP chest radiography [9]. Although these studies integrated bronchoscopy and imaging in assessing pulmonary infections, they lacked quantification of these methods and relied on chest radiography over CT imaging for predicting MP outcomes. The current study demonstrated that the combined prediction using BS and CT score was more effective than a single prediction method, achieving an AUC of 0.824 and a sensitivity of 0.89. This indicates high reliability in predicting RMPP. However, it is important to recognise that while this method is a valuable adjunct in clinical MP treatment, it cannot completely replace other laboratory indicators in the assessment of airway infections.

The study’s limitations include its retrospective nature and moderately small sample size. Future research will involve large-scale prospective studies to further validate the predictive utility of BS and CT score.

## Conclusion

This study is among the first to explore the potential value of combining CT scores with endoscopic BS for the clinical prediction of MP in children. The findings suggest that this combination method might offer a more accurate assessment and prediction of RMPP compared with using either modality alone. Consequently, this study potentially provides clinicians with a useful predictive tool during the treatment process that could facilitate more targeted treatment approaches for patients with MP.

## Data Availability

All data generated or analysed during this study are included in this article. Further enquiries can be directed to the corresponding author.
