# Correction: Predictive value of bronchoscopy combined with CT score for refractory mycoplasma pneumoniae pneumonia in children

**DOI:** 10.1186/s12890-024-03120-8

**Published:** 2024-07-19

**Authors:** Weihong Lu, Xiangtao Wu, Yali Xu, Tuanjie Wang, Aiju Xiao, Xixia Guo, Yuping Xu, Duoduo Li, Shujun Li

**Affiliations:** Department of Pediatrics, The First Affiliated Hospital of Xinxiang Medical University, No. 88 of Jiankang Road, Weihui, 453100 Henan province China

**BMC Pulmonary Medicine (2024) 24:251**

10.1186/s12890-024-02996-w

Following publication of the original article, the authors flagged that the author Duoduo Li had erroneously not been marked as a co-corresponding author. The corresponding authorship has now been corrected in the published article, and the corrected corresponding authorship may be seen in this erratum.

